# SARS-CoV-2 lineage dynamics in England from September to November 2021: high diversity of Delta sub-lineages and increased transmissibility of AY.4.2

**DOI:** 10.1186/s12879-022-07628-4

**Published:** 2022-07-27

**Authors:** Oliver Eales, Andrew J. Page, Leonardo de Oliveira Martins, Haowei Wang, Barbara Bodinier, David Haw, Jakob Jonnerby, Christina Atchison, The COVID-19 Genomics UK (COG-UK) Consortium, Samuel C. Robson, Thomas R. Connor, Nicholas J. Loman, Tanya Golubchik, Rocio T. Martinez Nunez, David Bonsall, Andrew Rambaut, Luke B. Snell, Rich Livett, Catherine Ludden, Sally Corden, Eleni Nastouli, Gaia Nebbia, Ian Johnston, Katrina Lythgoe, M. Estee Torok, Ian G. Goodfellow, Jacqui A. Prieto, Kordo Saeed, David K. Jackson, Catherine Houlihan, Dan Frampton, William L. Hamilton, Adam A. Witney, Giselda Bucca, Cassie F. Pope, Catherine Moore, Emma C. Thomson, Ewan M. Harrison, Colin P. Smith, Fiona Rogan, Shaun M. Beckwith, Abigail Murray, Dawn Singleton, Kirstine Eastick, Liz A. Sheridan, Paul Randell, Leigh M. Jackson, Cristina V. Ariani, Sónia Gonçalves, Derek J. Fairley, Matthew W. Loose, Joanne Watkins, Samuel Moses, Sam Nicholls, Matthew Bull, Roberto Amato, Darren L. Smith, David M. Aanensen, Jeffrey C. Barrett, Dinesh Aggarwal, James G. Shepherd, Martin D. Curran, Surendra Parmar, Matthew D. Parker, Catryn Williams, Sharon Glaysher, Anthony P. Underwood, Matthew Bashton, Nicole Pacchiarini, Katie F. Loveson, Matthew Byott, Alessandro M. Carabelli, Kate E. Templeton, Thushan I. de Silva, Dennis Wang, Cordelia F. Langford, John Sillitoe, Rory N. Gunson, Simon Cottrell, Justin O’Grady, Dominic Kwiatkowski, Patrick J. Lillie, Nicholas Cortes, Nathan Moore, Claire Thomas, Phillipa J. Burns, Tabitha W. Mahungu, Steven Liggett, Angela H. Beckett, Matthew T. G. Holden, Lisa J. Levett, Husam Osman, Mohammed O. Hassan-Ibrahim, David A. Simpson, Meera Chand, Ravi K. Gupta, Alistair C. Darby, Steve Paterson, Oliver G. Pybus, Erik M. Volz, Daniela de Angelis, David L. Robertson, Inigo Martincorena, Louise Aigrain, Andrew R. Bassett, Nick Wong, Yusri Taha, Michelle J. Erkiert, Michael H. Spencer Chapman, Rebecca Dewar, Martin P. McHugh, Siddharth Mookerjee, Stephen Aplin, Matthew Harvey, Thea Sass, Helen Umpleby, Helen Wheeler, James P. McKenna, Ben Warne, Joshua F. Taylor, Yasmin Chaudhry, Rhys Izuagbe, Aminu S. Jahun, Gregory R. Young, Claire McMurray, Clare M. McCann, Andrew Nelson, Scott Elliott, Hannah Lowe, Anna Price, Matthew R. Crown, Sara Rey, Sunando Roy, Ben Temperton, Sharif Shaaban, Andrew R. Hesketh, Kenneth G. Laing, Irene M. Monahan, Judith Heaney, Emanuela Pelosi, Siona Silviera, Eleri Wilson-Davies, Helen Fryer, Helen Adams, Louis du Plessis, Rob Johnson, William T. Harvey, Joseph Hughes, Richard J. Orton, Lewis G. Spurgin, Yann Bourgeois, Chris Ruis, Áine O’Toole, Marina Gourtovaia, Theo Sanderson, Christophe Fraser, Jonathan Edgeworth, Judith Breuer, Stephen L. Michell, John A. Todd, Michaela John, David Buck, Kavitha Gajee, Gemma L. Kay, Sharon J. Peacock, David Heyburn, Katie Kitchman, Alan McNally, David T. Pritchard, Samir Dervisevic, Peter Muir, Esther Robinson, Barry B. Vipond, Newara A. Ramadan, Christopher Jeanes, Danni Weldon, Jana Catalan, Neil Jones, Ana da Silva Filipe, Chris Williams, Marc Fuchs, Julia Miskelly, Aaron R. Jeffries, Karen Oliver, Naomi R. Park, Amy Ash, Cherian Koshy, Magdalena Barrow, Sarah L. Buchan, Anna Mantzouratou, Gemma Clark, Christopher W. Holmes, Sharon Campbell, Thomas Davis, Ngee Keong Tan, Julianne R. Brown, Kathryn A. Harris, Stephen P. Kidd, Paul R. Grant, Li Xu-McCrae, Alison Cox, Pinglawathee Madona, Marcus Pond, Paul A. Randell, Karen T. Withell, Cheryl Williams, Clive Graham, Rebecca Denton-Smith, Emma Swindells, Robyn Turnbull, Tim J. Sloan, Andrew Bosworth, Stephanie Hutchings, Hannah M. Pymont, Anna Casey, Liz Ratcliffe, Christopher R. Jones, Bridget A. Knight, Tanzina Haque, Jennifer Hart, Dianne Irish-Tavares, Eric Witele, Craig Mower, Louisa K. Watson, Jennifer Collins, Gary Eltringham, Dorian Crudgington, Ben Macklin, Miren Iturriza-Gomara, Anita O. Lucaci, Patrick C. McClure, Matthew Carlile, Nadine Holmes, Christopher Moore, Nathaniel Storey, Stefan Rooke, Gonzalo Yebra, Noel Craine, Malorie Perry, Nabil-Fareed Alikhan, Stephen Bridgett, Kate F. Cook, Christopher Fearn, Salman Goudarzi, Ronan A. Lyons, Thomas Williams, Sam T. Haldenby, Jillian Durham, Steven Leonard, Robert M. Davies, Rahul Batra, Beth Blane, Moira J. Spyer, Perminder Smith, Mehmet Yavus, Rachel J. Williams, Adhyana I. K. Mahanama, Buddhini Samaraweera, Sophia T. Girgis, Samantha E. Hansford, Angie Green, Charlotte Beaver, Katherine L. Bellis, Matthew J. Dorman, Sally Kay, Liam Prestwood, Shavanthi Rajatileka, Joshua Quick, Radoslaw Poplawski, Nicola Reynolds, Andrew Mack, Arthur Morriss, Thomas Whalley, Bindi Patel, Iliana Georgana, Myra Hosmillo, Malte L. Pinckert, Joanne Stockton, John H. Henderson, Amy Hollis, William Stanley, Wen C. Yew, Richard Myers, Alicia Thornton, Alexander Adams, Tara Annett, Hibo Asad, Alec Birchley, Jason Coombes, Johnathan M. Evans, Laia Fina, Bree Gatica-Wilcox, Lauren Gilbert, Lee Graham, Jessica Hey, Ember Hilvers, Sophie Jones, Hannah Jones, Sara Kumziene-Summerhayes, Caoimhe McKerr, Jessica Powell, Georgia Pugh, Sarah Taylor, Alexander J. Trotter, Charlotte A. Williams, Leanne M. Kermack, Benjamin H. Foulkes, Marta Gallis, Hailey R. Hornsby, Stavroula F. Louka, Manoj Pohare, Paige Wolverson, Peijun Zhang, George MacIntyre-Cockett, Amy Trebes, Robin J. Moll, Lynne Ferguson, Emily J. Goldstein, Alasdair Maclean, Rachael Tomb, Igor Starinskij, Laura Thomson, Joel Southgate, Moritz U. G. Kraemer, Jayna Raghwani, Alex E. Zarebski, Olivia Boyd, Lily Geidelberg, Chris J. Illingworth, Chris Jackson, David Pascall, Sreenu Vattipally, Timothy M. Freeman, Sharon N. Hsu, Benjamin B. Lindsey, Keith James, Kevin Lewis, Gerry Tonkin-Hill, Jaime M. Tovar-Corona, MacGregor Cox, Khalil Abudahab, Mirko Menegazzo, Ben E. W. Taylor MEng, Corin A. Yeats, Afrida Mukaddas, Derek W. Wright, Rachel Colquhoun, Verity Hill, Ben Jackson, J. T. McCrone, Nathan Medd, Emily Scher, Jon-Paul Keatley, Tanya Curran, Sian Morgan, Patrick Maxwell, Ken Smith, Sahar Eldirdiri, Anita Kenyon, Alison H. Holmes, James R. Price, Tim Wyatt, Alison E. Mather, Timofey Skvortsov, John A. Hartley, Martyn Guest, Christine Kitchen, Ian Merrick, Robert Munn, Beatrice Bertolusso, Jessica Lynch, Gabrielle Vernet, Stuart Kirk, Elizabeth Wastnedge, Rachael Stanley, Giles Idle, Declan T. Bradley, Jennifer Poyner, Matilde Mori, Owen Jones, Victoria Wright, Ellena Brooks, Carol M. Churcher, Mireille Fragakis, Katerina Galai, Andrew Jermy, Sarah Judges, Georgina M. McManus, Kim S. Smith, Elaine Westwick, Stephen W. Attwood, Frances Bolt, Alisha Davies, Elen De Lacy, Fatima Downing, Sue Edwards, Lizzie Meadows, Sarah Jeremiah, Nikki Smith, Luke Foulser, Themoula Charalampous, Amita Patel, Louise Berry, Tim Boswell, Vicki M. Fleming, Hannah C. Howson-Wells, Amelia Joseph, Manjinder Khakh, Michelle M. Lister, Paul W. Bird, Karlie Fallon, Thomas Helmer, Claire L. McMurray, Mina Odedra, Jessica Shaw, Julian W. Tang, Nicholas J. Willford, Victoria Blakey, Veena Raviprakash, Nicola Sheriff, Lesley-Anne Williams, Theresa Feltwell, Luke Bedford, James S. Cargill, Warwick Hughes, Jonathan Moore, Susanne Stonehouse, Laura Atkinson, Jack C. D. Lee, Divya Shah, Adela Alcolea-Medina, Natasha Ohemeng-Kumi, John Ramble, Jasveen Sehmi, Rebecca Williams, Wendy Chatterton, Monika Pusok, William Everson, Anibolina Castigador, Emily Macnaughton, Kate El Bouzidi, Temi Lampejo, Malur Sudhanva, Cassie Breen, Graciela Sluga, Shazaad S. Y. Ahmad, Ryan P. George, Nicholas W. Machin, Debbie Binns, Victoria James, Rachel Blacow, Lindsay Coupland, Louise Smith, Edward Barton, Debra Padgett, Garren Scott, Aidan Cross, Mariyam Mirfenderesky, Jane Greenaway, Kevin Cole, Phillip Clarke, Nichola Duckworth, Sarah Walsh, Kelly Bicknell, Robert Impey, Sarah Wyllie, Richard Hopes, Chloe Bishop, Vicki Chalker, Ian Harrison, Laura Gifford, Zoltan Molnar, Cressida Auckland, Cariad Evans, Kate Johnson, David G. Partridge, Mohammad Raza, Paul Baker, Stephen Bonner, Sarah Essex, Leanne J. Murray, Andrew I. Lawton, Shirelle Burton-Fanning, Brendan A. I. Payne, Sheila Waugh, Andrea N. Gomes, Maimuna Kimuli, Darren R. Murray, Paula Ashfield, Donald Dobie, Fiona Ashford, Angus Best, Liam Crawford, Nicola Cumley, Megan Mayhew, Oliver Megram, Jeremy Mirza, Emma Moles-Garcia, Benita Percival, Megan Driscoll, Leah Ensell, Helen L. Lowe, Laurentiu Maftei, Matteo Mondani, Nicola J. Chaloner, Benjamin J. Cogger, Lisa J. Easton, Hannah Huckson, Jonathan Lewis, Sarah Lowdon, Cassandra S. Malone, Florence Munemo, Manasa Mutingwende, Roberto Nicodemi, Olga Podplomyk, Thomas Somassa, Andrew Beggs, Alex Richter, Claire Cormie, Joana Dias, Sally Forrest, Ellen E. Higginson, Mailis Maes, Jamie Young, Rose K. Davidson, Kathryn A. Jackson, Lance Turtle, Alexander J. Keeley, Jonathan Ball, Timothy Byaruhanga, Joseph G. Chappell, Jayasree Dey, Jack D. Hill, Emily J. Park, Arezou Fanaie, Rachel A. Hilson, Geraldine Yaze, Stephanie Lo, Safiah Afifi, Robert Beer, Joshua Maksimovic, Kathryn McCluggage, Karla Spellman, Catherine Bresner, William Fuller, Angela Marchbank, Trudy Workman, Ekaterina Shelest, Johnny Debebe, Fei Sang, Marina Escalera Zamudio, Sarah Francois, Bernardo Gutierrez, Tetyana I. Vasylyeva, Flavia Flaviani, Manon Ragonnet-Cronin, Katherine L. Smollett, Alice Broos, Daniel Mair, Jenna Nichols, Kyriaki Nomikou, Lily Tong, Ioulia Tsatsani, Prof Sarah O’Brien, Steven Rushton, Roy Sanderson, Jon Perkins, Seb Cotton, Abbie Gallagher, Elias Allara, Clare Pearson, David Bibby, Gavin Dabrera, Nicholas Ellaby, Eileen Gallagher, Jonathan Hubb, Angie Lackenby, David Lee, Nikos Manesis, Tamyo Mbisa, Steven Platt, Katherine A. Twohig, Mari Morgan, Alp Aydin, David J. Baker, Ebenezer Foster-Nyarko, Sophie J. Prosolek, Steven Rudder, Chris Baxter, Sílvia F. Carvalho, Deborah Lavin, Arun Mariappan, Clara Radulescu, Aditi Singh, Miao Tang, Helen Morcrette, Nadua Bayzid, Marius Cotic, Carlos E. Balcazar, Michael D. Gallagher, Daniel Maloney, Thomas D. Stanton, Kathleen A. Williamson, Robin Manley, Michelle L. Michelsen, Christine M. Sambles, David J. Studholme, Joanna Warwick-Dugdale, Richard Eccles, Matthew Gemmell, Richard Gregory, Margaret Hughes, Charlotte Nelson, Lucille Rainbow, Edith E. Vamos, Hermione J. Webster, Mark Whitehead, Claudia Wierzbicki, Adrienn Angyal, Luke R. Green, Max Whiteley, Emma Betteridge, Iraad F. Bronner, Ben W. Farr, Scott Goodwin, Stefanie V. Lensing, Shane A. McCarthy, Michael A. Quail, Diana Rajan, Nicholas M. Redshaw, Carol Scott, Lesley Shirley, Scott A. J. Thurston, Will Rowe, Amy Gaskin, Thanh Le-Viet, James Bonfield, Jennifier Liddle, Andrew Whitwham, Deborah Ashby, Wendy Barclay, Graham Taylor, Graham Cooke, Helen Ward, Ara Darzi, Steven Riley, Marc Chadeau-Hyam, Christl A. Donnelly, Paul Elliott

**Affiliations:** 1 School of Public Health, Imperial College London, Norfolk Place, London, W2 1PG UK; 2 MRC Centre for Global Infectious Disease Analysis and Jameel Institute, Imperial College London, London, UK; 3 Quadram Institute, Norwich, UK; 4 MRC Centre for Environment and Health, School of Public Health, Imperial College London, London, UK; 5 Department of Infectious Disease, Imperial College London, London, UK; 6 Imperial College Healthcare NHS Trust, London, UK; 7 National Institute for Health Research Imperial Biomedical Research Centre, London, UK; 8 Institute of Global Health Innovation, Imperial College London, London, UK; 9 Department of Statistics, University of Oxford, Oxford, UK; 10 Health Data Research (HDR) UK, Imperial College London, London, UK; 11 UK Dementia Research Institute Centre at Imperial, Imperial College London, London, UK

**Keywords:** SARS-CoV-2, COVID-19, Delta variant, Genetic diversity, Transmission advantage, Mutation

## Abstract

**Background:**

Since the emergence of SARS-CoV-2, evolutionary pressure has driven large increases in the transmissibility of the virus. However, with increasing levels of immunity through vaccination and natural infection the evolutionary pressure will switch towards immune escape. Genomic surveillance in regions of high immunity is crucial in detecting emerging variants that can more successfully navigate the immune landscape.

**Methods:**

We present phylogenetic relationships and lineage dynamics within England (a country with high levels of immunity), as inferred from a random community sample of individuals who provided a self-administered throat and nose swab for rt-PCR testing as part of the REal-time Assessment of Community Transmission-1 (REACT-1) study. During round 14 (9 September–27 September 2021) and 15 (19 October–5 November 2021) lineages were determined for 1322 positive individuals, with 27.1% of those which reported their symptom status reporting no symptoms in the previous month.

**Results:**

We identified 44 unique lineages, all of which were Delta or Delta sub-lineages, and found a reduction in their mutation rate over the study period. The proportion of the Delta sub-lineage AY.4.2 was increasing, with a reproduction number 15% (95% CI 8–23%) greater than the most prevalent lineage, AY.4. Further, AY.4.2 was less associated with the most predictive COVID-19 symptoms (p = 0.029) and had a reduced mutation rate (p = 0.050). Both AY.4.2 and AY.4 were found to be geographically clustered in September but this was no longer the case by late October/early November, with only the lineage AY.6 exhibiting clustering towards the South of England.

**Conclusions:**

As SARS-CoV-2 moves towards endemicity and new variants emerge, genomic data obtained from random community samples can augment routine surveillance data without the potential biases introduced due to higher sampling rates of symptomatic individuals.

**Supplementary Information:**

The online version contains supplementary material available at 10.1186/s12879-022-07628-4.

## Background

Since its first documented case in India in November 2020 [1] the Delta variant of SARS-CoV-2 has spread rapidly across the world and by 16 November 2021 was responsible for 99.7% of all SARS-CoV-2 infections [2]. Its rapid rise to dominance has been attributed to greater levels of transmissibility [3, 4] than previously circulating variants with the reproduction number estimated to be over two-fold higher [5], as well as possible reduced vaccine effectiveness against infection [6]. Since its global dissemination, continued adaptive evolution has led to a diverse set of Delta sub-lineages, with distinct combinations of mutations (especially on the spike protein) [7, 8].

Since July 2021 the lineage AY.4.2 (Pango nomenclature [9]), a descendant of the original Delta variant (henceforth B.1.617.2) has increased in proportion in routine surveillance data for England from 8.5% the week beginning 4 October [10] to 14.7% the week beginning 31 October [11]. AY.4.2 was declared a variant under investigation (VUI) by the UK Health Security Agency on 20 October 2021 [12]. Globally AY.4.2 had been detected in 43 countries by 22 November 2021 [13] but had only been estimated at a cumulative proportion greater than 1% in Poland [14]. AY.4.2 has two defining mutations in the spike protein, Y145H and A222V, but is otherwise similar to AY.4, a lineage that is far more widespread. AY.4 was the most prevalent lineage in England on 29 October 2021 [11] and had been detected in 87 countries by 22 November 2021 [15], in some of which it had already been reported as the most prevalent lineage (by 23 November 2021) [16, 17].

England has recorded high levels of SARS-CoV-2 infection over the course of the pandemic [4, 18] and vaccinated, as part of its mass vaccination campaign (Pfizer/BioNTec, Oxford/AstraZeneca and Moderna), a large proportion of its population (80.3% of over 12 year olds double vaccinated by 27 November 2021), with further booster jabs (Pfizer/BioNTec or Moderna) being rolled out in adults (30.5% of over 12 year olds having received a booster dose by 27 November 2021) [18]. This has led to high levels of antibodies against coronavirus with 92.8% of adults in England estimated to test positive for antibodies (IgG antibodies against the SARS-CoV-2 trimeric spike protein) in the week beginning 1 November 2021 [19]. With high vaccination coverage in the population it is likely that there is substantial selective pressure on SARS-CoV-2 towards immune escape and vaccine breakthrough infections. Genomic surveillance in highly immunised regions is crucial to detect emerging variants that can more successfully navigate the immune landscape that has been created by both natural infection and vaccination.

The REal-time Assessment of Community Transmission-1 (REACT-1) study is a series of cross-sectional surveys of the population of England that seeks to estimate the prevalence of SARS-CoV-2 on a monthly basis [4, 20], with genomic sequencing performed on all positive samples with a low enough cycle threshold (Ct) value (a proxy for viral load) and high enough volume. Due to its sampling procedure it does not suffer from the biases of routine surveillance that can be heavily biased towards symptomatic individuals [21]; symptom status can be highly dependent on levels of immunity [22]. Here we present the genomic analysis of the (N = 2163) positive samples for round 14 and round 15 which were collected from 9 to 27 September 2021 and 19 October to 5 November 2021 respectively.

## Material and methods

### Viral genome sequencing

The methods of the REACT-1 study have been described elsewhere [23]. REACT-1 is a repeat cross-sectional study whereby in each round a random subset of the English population (selected from the National Health Service general practitioners' patient list) is invited to obtain a self-administered swab test (parent/guardian administered for 5–12 year olds). These tests are then sent to a laboratory to undergo rt-PCR testing for the presence of SARS-CoV-2. A round of the study covers a ~ 2- to 3-week period and has occurred approximately monthly since May 2020 with between 100,000 and 185,000 individuals taking part in each round. Since round 8 in January 2021 all positive samples with a low enough N-gene Ct value (the threshold was 34 in rounds 14 and 15 presented here) and sufficient volume have been sent for genome sequencing. Amplification of the extracted RNA was performed using the ARTIC protocol [24] (version 4 primers), with sequence libraries prepared using CoronaHiT [25]; sequencing was performed on the Illumina NextSeq 500 platform. Raw sequences were analysed using the bioinformatic pipeline [26] and then uploaded to CLIMB [27]. Lineages were assigned using PangoLEARN [28] (database version 2021-11-04), a machine learning-based assignment algorithm, using Pango nomenclature [9]. For some sequences of low overall quality, a lineage designation was not possible and so they were not included in the analyses. Samples with less than 50% of bases covered were further excluded from the analysis. Of the 1322 lineages determined during rounds 14 and 15, 1160 individuals provided information on their symptoms in the previous month with 314 (27.1%) reporting no symptoms.

### Phylogeographic model

For all sequences from REACT-1 rounds 11 (15 April–3 May 2021), 12 (20 May–7 June 2021), 13 (24 June–12 July 2021), 14 (9 September–27 September 2021) and 15 (19 October–5 November 2021), in which the lineage designated was Delta or a Delta sub-lineage, a maximum likelihood phylogenetic tree was constructed using a HKY model implemented in IQ-TREE [29]. An uncorrelated relaxed clock model implemented in TreeTime [30], assuming a normal distribution of rates with mean 0.0008 substitutions per site per year and a single coalescent rate for the time scale, was then fit to the maximum likelihood phylogenetic tree producing a time-resolved phylogenetic tree. The mutation rates at the tree’s tips were extracted from the model and a Gaussian regression model was fit to the samples obtained during round 14 and 15 for the 8 most prevalent lineages (AY.39, AY.4, AY.4.2, AY.43, AY.44, AY.5, AY.6, B.1.617.2) including lineage and round as covariates. A mugration model (implemented in TreeTime [30]) was run on the time-resolved phylogenetic tree, treating the region in which each sample was isolated as a discrete state. This allowed estimates of the migration rates between regions to be calculated (assumed to be symmetric).

### Statistical analyses

The 95% confidence intervals for lineage proportions were calculated using the Wilson method [31] assuming a Binomial distribution. This method is preferred when the number of positives is low but is still valid when this is not the case [32]. Higher accuracy in confidence interval estimates for when the number of positives is low was chosen so that lower bounds on case numbers for rarer lineages were as accurate as possible.

Estimates of the true number of swab-positive infections in England during round 14 and round 15 for lineages in which only one sample was detected in a round were calculated by multiplying the estimated proportion of the lineage for each round, the weighted prevalence estimated for each round [33], and the population size of England [34]. The 95% confidence intervals were estimated by simulating the entire distribution for proportion and weighted prevalence and multiplying the two together. The distribution of weighted prevalence was estimated by randomly sampling from a normal distribution with mean value the central estimate, and standard deviation the width of the 95% confidence interval divided by 3.92 (2 times 1.96). The distribution of the lineage proportion was estimated by calculating the Wilson confidence intervals at different levels (0.00001 to 0.99999 in intervals of 0.00001).

The significance of differences in proportions of particular lineages by age group and region was calculated using Fisher’s exact test with a binary outcome variable (lineage of interest or not). Differences with a p-value less than 0.05 were considered statistically significant. Analysis was only completed for a lineage in a round if there were more than 90 samples (AY.4 round 14, AY.4 round 15, AY.4.2 round 15, B.1.617.2 round 15), so that there were, on average, more than 10 samples per parameter (9 regions in England).

Shannon diversity was calculated using all data for round 14 and round 15, and for each region for round 14 and round 15 [35]. The significance of any differences in Shannon diversity between round 14 and 15 (for all data) and between regions in each round was assessed using the Hutcheson T-test [36] and its associated p-value.

The relative growth rate of a lineage compared to all other lineages was estimated using a Bayesian logistic regression model fit to the binary outcome variable (lineage of interest or not) over time. The two model parameters (intercept and gradient) were given uninformative constant prior distributions. The probability that the growth rate was greater than zero was calculated from the model's posterior. Lineages were deemed to be different to zero if the posterior probability that the growth rate was greater than zero was greater than 0.975 or less than 0.025, similar to a p-value threshold of 0.05.

The growth rates of AY.4.2 and AY.4 infected individuals were estimated by fitting an exponential model to the daily weighted prevalence using all REACT-1 data (all negatives and all AY.4/AY.4.2 associated positives) for rounds 14 and 15 assuming a Binomial likelihood. Weightings for individual REACT-1 samples were calculated using rim weighting [37] by: sex, deciles of the IMD, LTLA counts and ethnic group. Growth rates were then converted to estimates of the reproduction number R assuming a gamma-distributed generation time with the shape parameter, n = 2.29, and rate parameter, b = 0.36 [38] through the equation $(1 + \frac{r}{b}{)}^{n}$ [39]. The multiplicative R advantage of AY.4.2 over AY.4 was estimated using the entire posterior distribution of ${R}_{AY.4.2}/{R}_{AY.4}$ with the median and 95% credible interval reported.

For each lineage with more than 1 sample in a round the presence of clustering was assessed. The pairwise distance matrix between all n samples that were designated to a specific lineage was calculated and from this a mean pairwise distance was calculated for the lineage. Next, 10,000 random combinations of n positive individuals (n positive individuals chosen each time without replacement), for which any lineage was determined, were selected and for each combination the distance matrix and mean distance was calculated. The proportion of the 10,000 estimated mean distances below the lineage-specific mean distance was then calculated. Clustering was deemed to be significant if this proportion was less than 0.05.

For the 8 most prevalent lineages across rounds 14 and 15 Gaussian regression was performed to estimate the mean N- and E-gene Ct values for each lineage and p-values used to assess the significance of any difference to the reference lineage (AY.4). Models were run on all data (rounds 14 and 15 combined) and then run on data from each individual round as a sensitivity analysis.

The proportion of individuals reporting any symptoms in the month prior to swabbing and any of the most predictive COVID-19 symptoms in the month prior to swabbing was calculated for the 8 most prevalent lineages across rounds 14 and 15. P-values were estimated for each lineage relative to AY.4 by performing logistic regression with the symptom status as a binary variable (any symptoms vs no symptoms, and separately most predictive COVID-19 symptoms vs none of the most predictive COVID-19 symptoms). The sensitivity of the results that AY.4.2 is less likely to exhibit the most predictive COVID-19 symptoms, relative to AY.4, was assessed by fitting further logistic regression models including age, round of study and N-gene Ct value as covariates (E-gene was also investigated but was no different to using N-gene and so this was not included).

## Results

Lineage diversity.

**Fig. 1:**
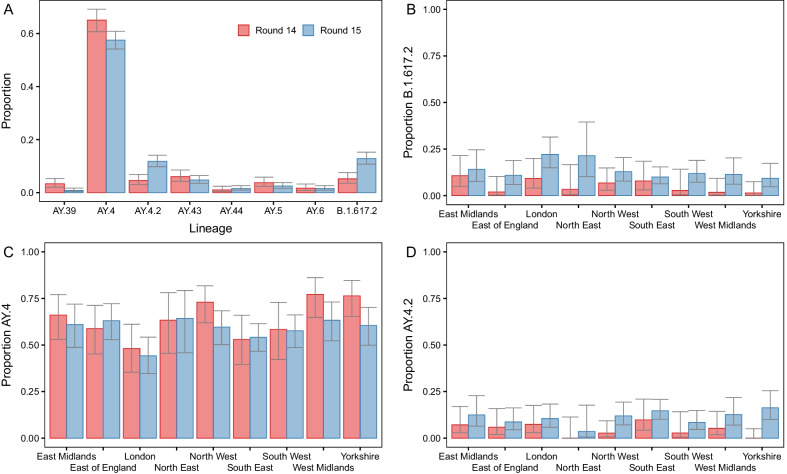
Proportion of positives by Delta sub-lineage. **A** The proportion of positives samples by round designated to the 8 lineages most prevalent over both rounds 14 and 15 (AY.39, AY.4, AY.4.2, AY.43, AY.44, AY.5, AY.6, B.1.617.2). **B**–**D** Proportion of positive samples by round and region with lineage designated as **B** B.1.617.2, **C** AY.4 and **D** AY.4.2. For all figures, point estimates of proportion are shown (bars) with 95% confidence intervals (error-bars)

In round 14 the lineage was determined for 481 of 764 positive samples. All lineages were Delta or a Delta sub-lineage with the four most prevalent lineages being AY.4 at 65.1% (60.7%, 69.2%, n = 313), AY.43 at 6.0% (4.2%, 8.5%, n = 29), B.1.617.2 (original Delta variant) at 5.2% (3.6%, 7.6%, n = 25) and AY.4.2 at 4.6% (3.0%, 6.8%, n = 22) (Fig. 1-A, Additional file 2: Table S1). In round 15 the lineage was determined for 841 of 1399 positive samples. Again all samples were Delta or a Delta sub-lineage with the most prevalent lineages again being AY.4 at 57.6% (54.2%, 60.9%, n = 484), B.1.617.2 at 12.8% (10.8%, 15.3%, n = 108), AY.4.2 at 11.8% (9.8%, 14.1%, n = 99) and AY.43 at 4.8% (3.5%, 6.4%, n = 40). The next four most prevalent lineages over both rounds combined were AY.5, AY.6, AY.39, and AY.44. However, even a single detection of a lineage corresponded nationally to an average of 971 (95% CI [171, 5463]) individuals that would test swab-positive on any given day during round 14 and 1051 (95% CI [185, 5928]) individuals that would test swab-positive on any given day during round 15. During rounds 14 and 15 there were 33 and 31 unique lineages detected, respectively with 44 unique lineages detected overall. There was no apparent difference in genetic diversity between the two rounds as estimated by the Shannon diversity (p = 0.831) (Additional file 2: Table S2).

### Distribution by region and age

During round 15 the proportion of B.1.617.2 was found to be highest in London at 22.1% (14.9%, 31.4%), being greater than the proportion in South East, East of England and Yorkshire and The Humber (Fig. 1B, Additional file 2: Table S3). Conversely, in round 14 and 15 the proportion of AY.4 was lowest in London at 48.1% (35.4%, 61.1%) and 44.2% (34.6%, 54.2%) respectively and was found to be higher in North West, West Midlands and Yorkshire and The Humber during both rounds (Fig. 1C, Additional file 2: Table S3). This reduced proportion of the nationally most prevalent lineage (AY.4) in London coincided with a higher level of genetic diversity in London. The Shannon diversity was highest in London during both rounds at 1.814 in round 14 and 1.809 in round 15 (p < 0.001 and p = 0.002 respectively, reference = West Midlands, Additional file 2: Table S2). Higher levels of genetic diversity were also found during both rounds in the South East and South West, relative to the West Midlands (which showed the lowest levels of genetic diversity in round 14 and the second lowest in round 15). There were no regional differences in the proportion of AY.4.2 during round 15 (Fig. 1D, Additional file 2: Table S3). Regional differences during round 14 and regional differences for other lineages could not be investigated due to small sample sizes but numbers are provided in Additional file 2: Table S4.

**Fig. 2:**
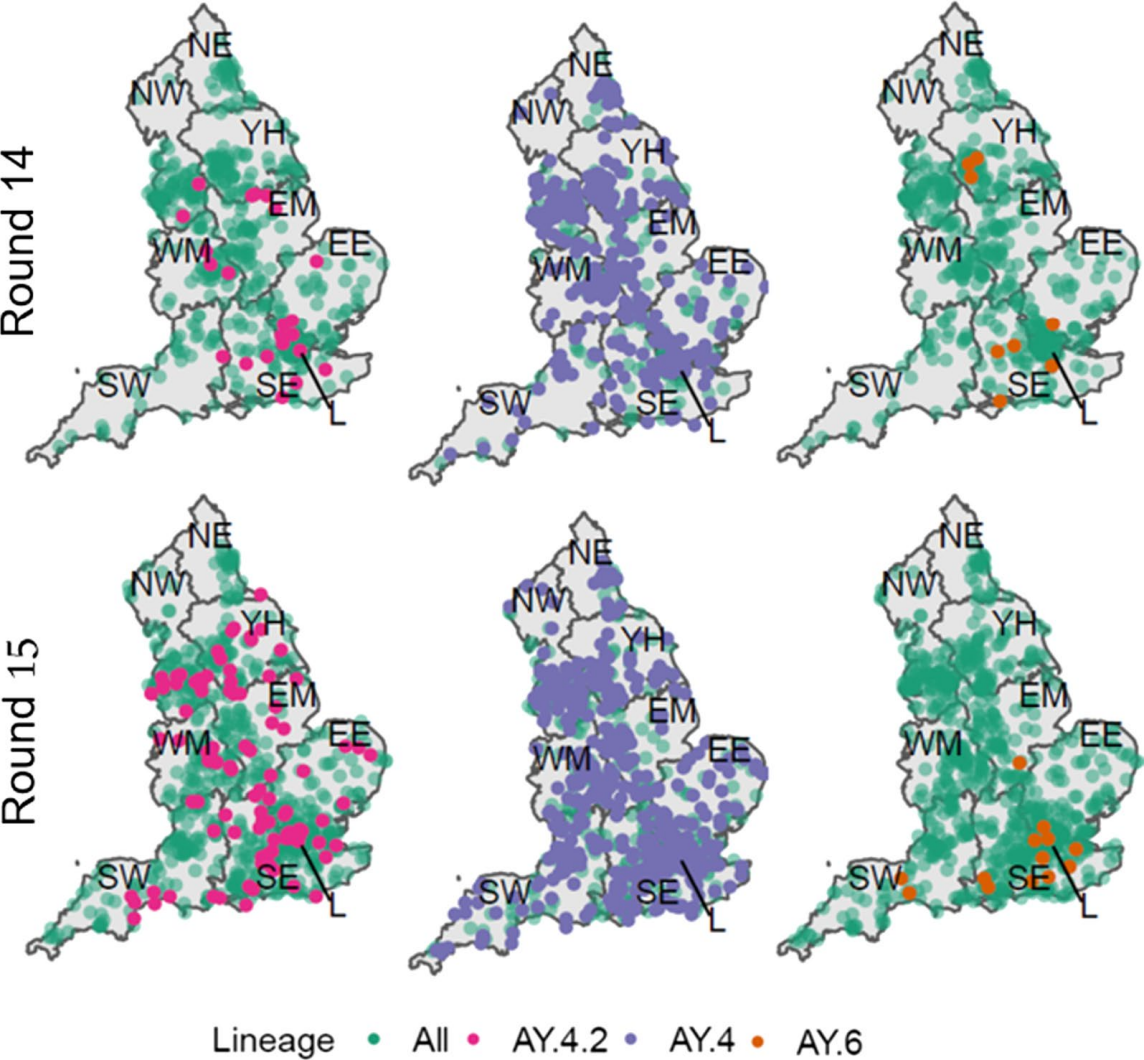
Geographic distribution of all positive samples with a lineage designation (Green) with overlaid distribution of AY.4.2 (Pink, left), AY.4 (Purple, centre) and AY.6 (Orange, right) for both round 14 (top) and round 15 (bottom). The lineages shown had either a significant level of clustering in round 14 (AY.4 and AY.4.2) or round 15 (AY.6)

Sub-regional analysis was performed in order to investigate the presence of clustering in each round for each lineage (see Methods). Despite being highly geographically dispersed (Fig. 2) clustering was detected in round 14 for AY.4 (p = 0.037) and AY.4.2 (p = 0.029) (Additional file 2: Table S5). However, during round 15 clustering was no longer evident for both AY.4 (p = 0.706) and AY.4.2 (p = 0.067). The only lineage for which clustering was detected in round 15 was AY.6 (p = 0.003) which was found mainly in London and towards the South coast of England.

During round 15 the proportion of B.1.617.2 was higher in individuals ages 25–34 years old at 24.2% (12.8%, 41.0%) relative to those aged 35–44 years old at 8.0% (4.1%, 15.0%) (p = 0.026) (Additional file 2: Table S6). The proportion of AY.4 was found to be lower in 5–12 year olds at 52.1% (44.6%, 59.5%) relative to 35–44 year olds in which the proportion of AY.4 was 65.0% (55.3%, 73.6%) (p = 0.042) in round 15, while it was not in round 14.There were no differences between age groups in the proportion of AY.4.2 during round 15. Differences between age groups during round 14 for AY.4.2 and other lineages could not be investigated due to small sample sizes but numbers are provided in Additional file 2: Table S7.

### Detection of increasing sub-lineages

**Fig. 3:**
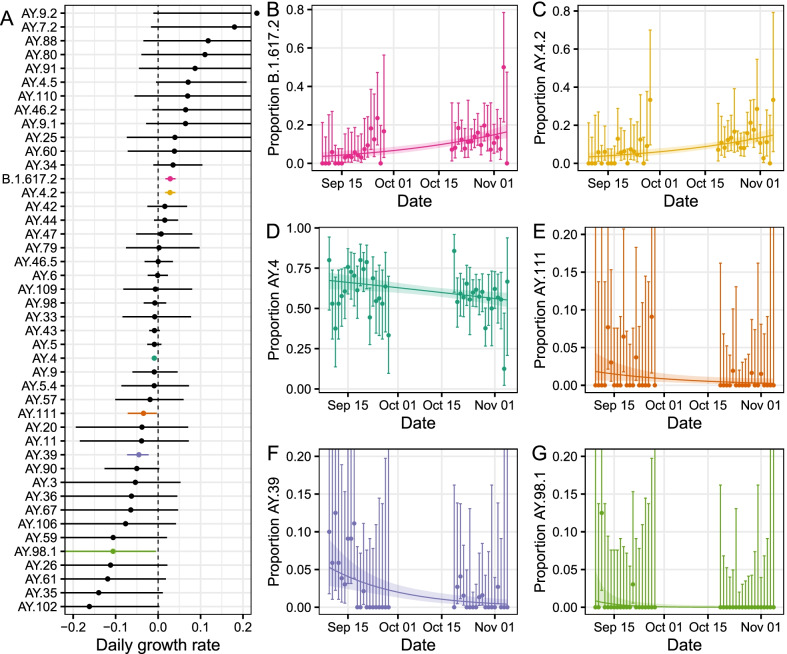
**A** Estimated daily growth rate of the log odds of each lineage detected relative to all other lineages. Shown are both lineages with a growth rate in proportion not significantly different to zero (black) and those with a growth rate in proportion significantly different to zero (coloured). **B**–**G** Raw estimates of the daily proportions (points) with 95% confidence intervals (error bars) for lineages with a growth rate in proportion significantly different to zero: B.1.617.2 (**B**, pink), AY.4.2 (**C**, yellow), AY.4 (**D**, dark green), AY.111 (**E**, orange), AY.39 (**F**, purple), AY.98.1 (**G**, light green). Also shown is the best-fit Bayesian logistic regression model with central estimate (solid line) and 95% credible interval (shaded region)

Logistic regression models were fitted to the proportion of each lineage detected in either round 14 or 15, allowing daily growth rates in proportion to be estimated (Fig. 3, Additional file 2: Table S8). Of the 44 unique lineages detected, 6 were estimated to have growth rates different to zero. AY.4, AY.39, AY.98.1 and AY.111 were decreasing in proportion, whereas AY.4.2 and B.1.617.2 were increasing in proportion. The decrease in proportion of AY.4 corresponded to a daily growth rate of − 0.009 (− 0.015, − 0.003). The increase in proportions of B.1.617.2 and AY.4.2 corresponded to growth rates of 0.029 (0.017, 0.041) and 0.028 (0.016, 0.041) respectively.

Comparing estimates of the reproduction number R from round 14 to round 15 for AY.4 and AY.4.2 (see Methods) we estimate a multiplicative R advantage of 1.15 (1.08, 1.23), assuming no change in the generation time distribution.

### Differences in cycle threshold values

**Fig. 4:**
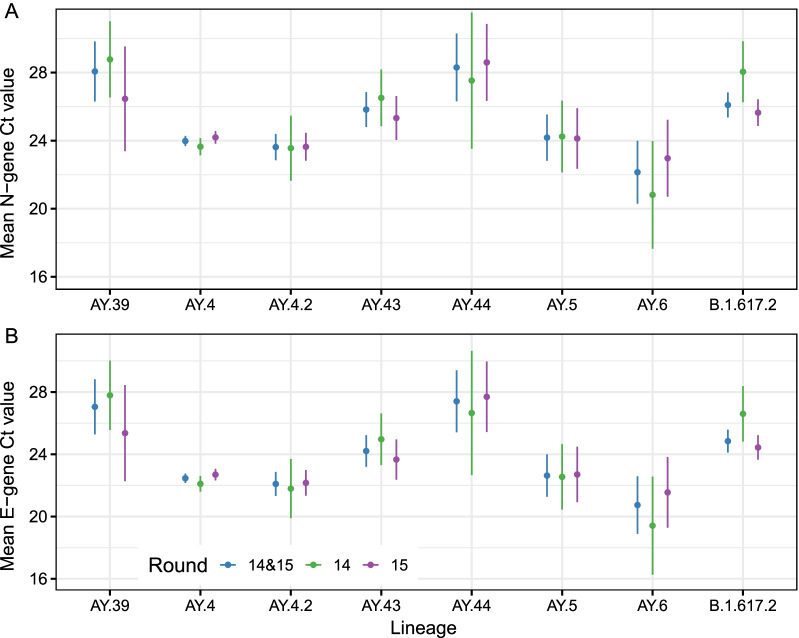
Estimated mean N-gene (**A**) and E-gene (**B**) Ct values for the 8 lineages most prevalent over rounds 14 and 15 (AY.39, AY.4, AY.4.2, AY.43, AY.44, AY.5, AY.6 and B.1.617.2) as calculated using Gaussian regression. Point estimates (points) and 95% confidence intervals (lines) are shown for estimates obtained using data from both rounds (blue), data from just round 14 (green) and data from just round 15 (purple)

There were quantitative differences between lineages in the N- and E-gene Ct values. The mean N- and E-gene Ct values were lowest for AY.6 though not materially lower than the values obtained for AY.4 (Fig. 4, Additional file 2: Table S9). Mean N-gene Ct value was 22.14 (20.30, 23.99) for AY.6 compared to 23.98 (23.68, 24.28) for AY.4 (p = 0.054). Mean E-gene Ct value was 20.74 (18.90, 22.59) for AY.6 compared to 22.46 (22.16, 22.76) for AY.4 (p = 0.071). Mean N- and E-gene Ct values were found to be comparable to AY.4 for both AY.4.2 and AY.5. Relative to AY.4, mean N- and E-gene Ct values for AY.43, AY.44, AY.39 and B.1.617.2 were all higher.

### Differences in symptomatology

**Fig. 5:**
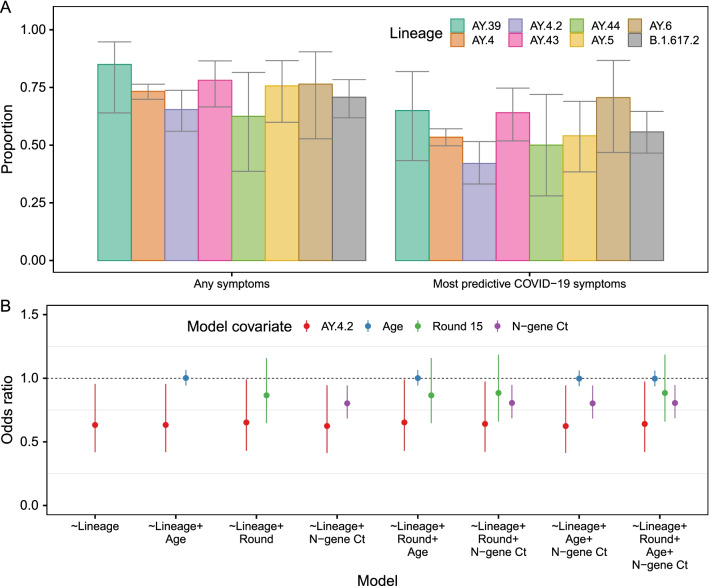
**A** Proportion of positive individuals reporting any symptoms or reporting one of the four most predictive COVID-19 symptoms (loss or change of sense of taste, loss or change of sense of smell, new persistent cough, fever) in the last month by lineage of infection, for the 8 lineages most prevalent during rounds 14 and 15 (AY.39, AY.4, AY.4.2, AY.43, AY.44, AY.5, AY.6 and B.1.617.2). Point estimates of proportion are shown (bars) with 95% confidence intervals (error-bars). **B** Odds ratios of reporting the most predictive COVID-19 symptoms in the last months for multivariable logistic regression models including lineage (AY.4.2 with reference AY.4, red), age (relative to change of 10 years in age, blue), round of study (round 15 with reference round 14, green) and N-gene Ct value (relative to change in Ct value of 5, purple). The central estimates of odds ratios are shown (points) with 95% confidence intervals (error-bars)

The proportion of individuals exhibiting the most predictive COVID-19 symptoms (loss or change of sense of taste, loss or change of sense of smell, new persistent cough, fever) in the month prior to swabbing was lower (p = 0.029) in those infected with AY.4.2 at 42.1% (33.1%, 51.5%) relative to those infected with AY.4 at 53.4% (49.7%, 57.1%) (Fig. 5A, Additional file 2: Table S10). This difference was not explained by patterns in age, round of the study or N-gene Ct value (Fig. 5B, Additional file 2: Table S11).

In addition, 68.6% (59.8%, 76.3%) of those infected with AY.4.2 reported any symptoms in the month prior to swabbing compared to 75.4% (72.2%, 78.3%) for those infected with AY.4 (p = 0.119). There were no differences evident in symptom reporting between AY.4 infected individuals and the other 6 most prevalent lineages (B.1.617.2, AY.5, AY.6, AY.43, AY.44 and AY.39).

### Phylogeographic analysis

**Fig. 6:**
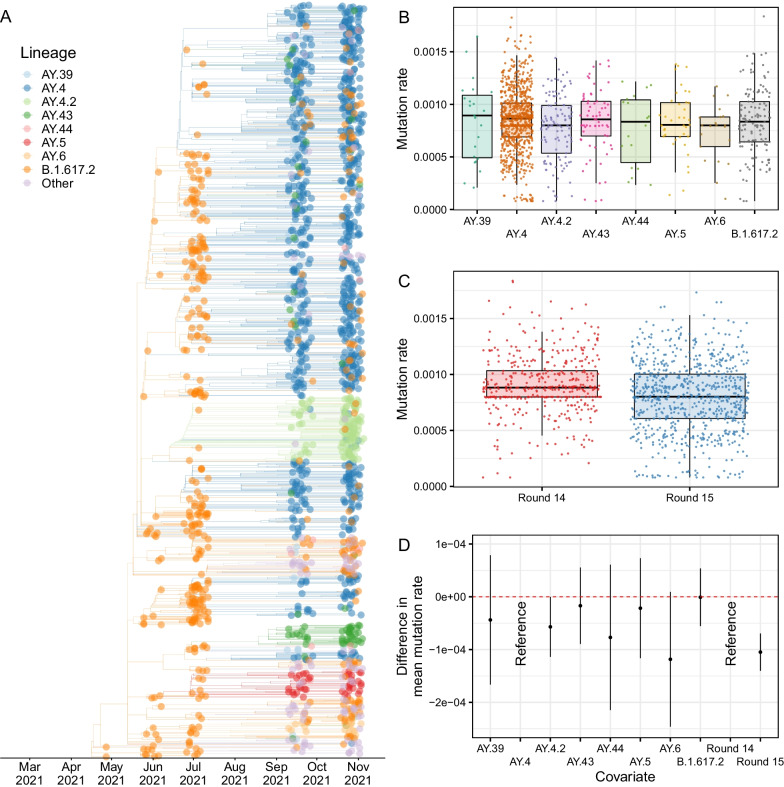
**A** Time-resolved phylogenetic tree of all positive samples obtained for which the lineage designated was Delta or a Delta sub-lineage. **B** Distribution of mutation rates inferred at each phylogenetic tree tip for the 8 lineages most prevalent in round 14 and round 15 for samples obtained in round 14 and 15. **C** Distribution of mutation rates inferred at each phylogenetic tree tip for samples collected in round 14 and round 15. In **B** and **C** the estimates for mutation rate at each tip are given (points), the median (horizontal line) and interquartile range (boxes) of each distribution, and the range of the maximum and minimum values that are within 1.5 times the interquartile range of the box (vertical line). **D** Difference in the mean mutation rates between lineages and rounds as inferred from a Gaussian regression model. Central estimates of the differences are shown (points) with 95% confidence intervals (error-bars)

A relaxed molecular clock model was fit to the data and used to estimate a time-resolved phylogenetic tree (Fig. 6). AY.4.2 was found to populate two closely related clades that emerged in June/July 2021. AY.43, AY.5 and AY.6 were also observed to have distinct clade groupings having emerged around June/July 2021 as well. The mutation rates inferred at the tree’s tips showed a large degree of variation in all of the 8 most prevalent lineages. The mean mutation rate for AY.4.2 was found to be 0.57 (< 0.01, 1.10)$\times 1{0}^{-4}$ lower than the mean mutation rate of AY.4 (p = 0.050) (Fig. 6, Additional file 2: Table S2). The mean mutation rate inferred for samples collected in round 15 was found to be 1.00 (0.70, 1.40)$\times 1{0}^{-4}$ lower than the mean mutation rate for samples collected in round 14 (p < 0.001).

A mugration model was run on the time-resolved phylogenetic tree to estimate the relative virus migration rates between regions, a measure of inter-region transmission (Additional file 2: Table S13). Overall levels of inter-region transmission were lowest for the North East during round 14 and 15. The highest overall level of inter-region transmission was observed for the North West during round 14 and 15, but looking at individual rounds there were higher levels for Yorkshire and The Humber in round 14 and for the South East in round 15. High rates of transmission during round 14 and 15 were found between the North West and Yorkshire and The Humber, the West Midlands and the South East, and also between the South East and London.

## Discussion

The proportion of AY.4.2 was found to be increasing between 9 September and 5 November 2021, as also reported in the routine data surveillance for England [11]. In round 15, AY.4.2 represented 11.8% of infections in line with other estimates [11]. This increase in proportion corresponded to a 15% increase in transmission advantage although this assumes the generation time distribution has remained constant; a decrease of the generation time distribution for AY.4.2 would also explain the increased growth but we are unable to test for this with prevalence data. In the past, the A222V mutation, associated with AY.4.2, increased in frequency but this was eventually deemed to be due to a founder effect and not a transmission advantage [40, 41]. Given the high levels of geographic dispersion (though with some clustering) during rounds 14 and 15 it is highly unlikely that a founder effect can explain the current growth, though we can not rule out a similar effect due to higher proportions of AY.4.2 in school-aged children (prevalence increased to a greater extent in school-aged children than in adults from July to September 2021 [4, 42]). However, as the proportion AY.4.2 was approximately constant by age in round 15 this growth advantage would not be detected into the future if this was the case.

Observed distributions of N- and E-gene Ct values were similar in AY.4.2 and AY.4 and so it is unlikely that the transmission advantage observed can be attributed to a higher viral load (a Ct 1 unit lower corresponds to an approximate twofold increase in viral load [43]). However, a reduced proportion of AY.4.2 infected individuals reporting symptoms could explain the increased transmissibility in multiple ways. Higher levels of asymptomatic infection could lead to greater levels of asymptomatic transmission. Further current testing procedures and government isolation advice in England heavily focus on the most predictive COVID-19 symptoms, which are reported less often by AY.4.2 infected individuals compared with AY.4. Thus, symptom-based policies could introduce an advantage for AY.4.2 over AY.4. Finally, the reduced level of symptom reporting could be indicative of greater levels of re-infection if AY.4.2 were more successful at evading the immune response. However, studies have found that vaccines are no less effective against AY.4.2 than other Delta sub-lineages [11] and vaccine-induced antibody neutralisation titres for AY.4.2 are similar to those for AY.4 and B.1.617.2 [44]. However, any possible evasion of the immune response caused by natural infections has yet to be investigated and the numbers reporting previous infection is too small and the proportion vaccinated too large in this REACT-1 dataset to allow a meaningful comparison (715 of 817 [87.5%] individuals aged 18 and over reported having had two vaccine doses). We found a moderately reduced mutation rate of AY.4.2 relative to AY.4 which may also have introduced a fitness advantage due to a smaller number of deleterious mutations [45, 46].

### Other lineages

Though we have focused on AY.4.2 we have detected a diverse set of Delta sub-lineages, with even a single detection corresponding to approximately 1000 swab-positive infections in the community at one time during the study period. The short time over which AY.4.2 went from being an undeclared lineage to a variant under investigation shows how crucial it is to have careful surveillance of all lineages irrespective of frequency. For 38 of the 44 detected lineages, it was unable to be determined whether the proportion was increasing or decreasing.

Between rounds 14 and 15 a reduction in the mean mutation rate of the virus was detected suggesting a reduction in the rate of evolution. However, despite this slowdown evolution is still occurring and we observed an increase in the proportion of B.1.617.2, an indicator that the number of undeclared B.1.617.2 sub-lineages was increasing, suggesting even further diversity of Delta sub-lineages that have yet to be given a unique lineage designation. Further, though we capture the dynamics within England, SARS-CoV-2 is a global problem and new variants of concern can arise anywhere in the world and then spread through international travel. Higher proportions of B.1.617.2 were detected in London as well as higher levels of diversity; this likely reflects the role London continues to play in the introduction of international variants [47]. Within England, the North West region played a major role in the dissemination of the virus, having the greatest inferred rate of inter-region transmission.

Analysis of N- and E-gene Ct values found decreased levels in AY.4 and AY.4.2, which is unsurprising given both have successfully disseminated across the country, but AY.5 and AY.6 were also found to have similarly low Ct values suggesting similar viral loads; the mean N- and E-gene Ct value appeared slightly lower for AY.6 compared to AY.4. Clustering was also detected in round 15 for AY.6; careful consideration of AY.6 should be given in the future in case the current lack of growth so far reported [11] has only been due to its geographic isolation.

### Limitations

We have presented the inferred dynamics between Delta sub-lineages in England between 9 September and 5 November 2021. Our sample's main strength over those obtained from routine surveillance is the random nature of the testing program leading to a relatively unbiased set of positive samples. However, as the sample sizes we obtain are relatively small compared with routine national surveillance our estimates have lower precision. Lineages were only successfully determined for ~ 61% of positive samples, with the ability to determine a lineage heavily influenced by a sample’s Ct value; this has potentially led to biases with lineages with lower Ct values more heavily represented in the dataset. Detecting distinct sub-lineages is a high-dimensional problem, with often many common mutations being shared between distinct lineages with only a small number of distinguishing mutations. This is exacerbated when all the lineages are highly related, as in the current nature of the pandemic in England where all samples are descendants of Delta (B.1.617.2), and can lead to incorrect designations [48]. Further, only sub-lineages that have been defined are able to be assigned to a sample. During the emergence of a new sub-lineage there is a phase of ambiguity when numbers are small and it is unclear if the mutations present warrant the declaration of a new sub-lineage. This can be seen in the detection of AY.4.2 and AY.43; both lineages had been circulating for months by October 2021 [11] but were not yet declared sub-lineages by pangoLEARN [28] in early October 2021, and so did not appear in the publicly available technical briefings [49]. The analysis of mutation rates using Gaussian regression may also have included biases as individual measurements of mutation rates would not have independent and identically distributed normal errors, a key assumption of these linear models.

## Conclusions

Since the beginning of the pandemic, selective pressure has led to rapid evolution in the spike protein [50] driving leaps in transmissibility [5]. However, as a greater proportion of the population acquires immunity through either infection or vaccination there will be a shift in evolutionary pressure towards immune escape. Even in England where there are high levels of vaccination and past infection, new variants such as AY.4.2 have emerged with advantages over previous strains. With the continued emergence of variants able to evade population immunity and undergoing transmission, SARS-CoV-2 is highly unlikely to ever undergo local extinction and is likely moving towards a state of endemicity. At the point of endemicity it is probable that adaptive evolution would more closely resemble the continual antigenic drift observed in influenza H3N2 [51, 52]. As the evolutionary phase of SARS-CoV-2 progresses towards endemicity, continued surveillance is paramount in not only detecting increased levels of transmissibility for specific lineages, but in also better characterising the mechanism behind such changes and informing policy around testing (including case definitions). Representative community studies such as REACT-1 can be useful in measuring the relative growth of lineages and in characterising differences in viral loads, symptomatology and geographic distribution.

## Supplementary Information

**Additional file 1.** REACT-1 sequence accession numbers for GISAID and the European Nucleotide Archive.

**Additional file 2: Table S1.** Lineages detected in rounds 14 and 15 of REACT-1. **Table S2.** Estimates of Shannon diversity for England, and by region for rounds 14 and 15 of REACT-1. **Table S3.** Regional distribution of AY.4 (round 14 and round 15), AY.4.2 (round 15) and B.1.617.2 (round 15). **Table S4.** Raw numbers of all lineages by region for round 14 and 15 of REACT-1. **Table S6.** Estimated P-value for the presence of clustering for all lineages with more than a single sample in an individual round, for round 14 and 15 of REACT-1. **Table S6.** Distribution of AY.4 (round 14 and round 15), AY.4.2 (round 15) and B.1.617.2 (round 15) by age group. **Table S7.** Raw numbers of all lineages by age group for round 14 and 15 of REACT-1. **Table S8.** Estimated growth rate in the log odds of every lineage detected relative to all other lineages from round 14 to 15 of REACT-1. **Table S9.** Mean N- and E-gene Ct value for the eight most prevalent lineages as inferred from Gaussian regression. **Table S10.** Symptom status by lineage for the eight most prevalent lineages in rounds 14 and 15 of REACT-1. **Table S11.** Multivariable logistic regression models to determine the effect of the lineage AY.4.2 on the odds of an individual reporting any of the most predictive COVID-19 symptoms relative to AY.4. **Table S12.** Multivariable gaussian regression model to determine the effect of lineage and round of the study on mean mutation rate. **Table S13.** Average inter-region migration rate, inferred from a mugration model run on a time-resolved phylogenetic tree, for the periods of rounds 14 and 15, round 14 and round 15

## Data Availability

Access to REACT-1 data is restricted due to ethical and security considerations. Summary statistics and descriptive tables from the current REACT-1 study are available in the Additional file 2. Additional summary statistics and results from the REACT-1 programme are also available at https://www.imperial.ac.uk/medicine/research-and-impact/groups/react-study/real-time-assessment-of-community-transmission-findings/ and https://github.com/mrc-ide/reactidd/tree/master/inst/extdata REACT-1 Study Materials are available for each round at https://www.imperial.ac.uk/medicine/research-and-impact/groups/react-study/react-1-study-materials/. Sequence read data are available without restriction from the European Nucleotide Archive at https://www.ebi.ac.uk/ena/browser/view/PRJEB37886, and consensus genome sequences are available from the Global initiative on sharing all influenza data at https://www.gisaid.org. Accession numbers are provided in the Additional file 1.
